# The diagnostic value of contrast-enhanced 2D mammography in everyday clinical use

**DOI:** 10.1038/s41598-021-01622-7

**Published:** 2021-11-15

**Authors:** L. M. F. H. Neeter, H. P. J. Raat, S. D. Meens-Koreman, R. S. A. van Stiphout, S. M. E. C. Timmermans, K. M. Duvivier, M. L. Smidt, J. E. Wildberger, P. J. Nelemans, M. B. I. Lobbes

**Affiliations:** 1 GROW School for Oncology and Developmental Biology, Maastricht University, Maastricht, The Netherlands; 2 Department of Radiology and Nuclear Medicine, Maastricht University Medical Center+, P.O. Box 5800, 6202 AZ Maastricht, The Netherlands; 3 Department of Medical Imaging, Laurentius Hospital, Roermond, The Netherlands; 4 Department of Radiology and Nuclear Medicine, Amsterdam UMC, Cancer Center Amsterdam, Vrije Universiteit Amsterdam, Amsterdam, The Netherlands; 5 Department of Surgery, Maastricht University Medical Center+, Maastricht, The Netherlands; 6 Department of Epidemiology, Maastricht University, Maastricht, The Netherlands; 7 Department of Medical Imaging, Zuyderland Medical Center, Sittard-Geleen, The Netherlands

**Keywords:** Medical imaging, Breast cancer

## Abstract

Contrast-enhanced mammography (CEM) has shown to be superior to full-field digital mammography (FFDM), but current results are dominated by studies performed on systems by one vendor. Information on diagnostic accuracy of other CEM systems is limited. Therefore, we aimed to evaluate the diagnostic performance of CEM on an alternative vendor’s system. We included all patients who underwent CEM in one hospital in 2019, except those with missing data or in whom CEM was used as response monitoring tool. Three experienced breast radiologists scored the low-energy images using the BI-RADS classification. Next, the complete CEM exams were scored similarly. Histopathological results or a minimum of one year follow-up were used as reference standard. Diagnostic performance and AUC were calculated and compared between low-energy images and the complete CEM examination, for all readers independently as well as combined. Breast cancer was diagnosed in 23.0% of the patients (35/152). Compared to low-energy images, overall CEM sensitivity increased from 74.3 to 87.6% (*p* < 0.0001), specificity from 87.8 to 94.6% (*p* = 0.0146). AUC increased from 0.872 to 0.957 (*p* = 0.0001). Performing CEM on the system tested, showed that, similar to earlier studies mainly performed on another vendor’s systems, both sensitivity and specificity improved when compared to FFDM.

## Introduction

Contrast-enhanced mammography (CEM) is an emerging breast imaging modality in which the intravenous administration of an iodine based contrast agent, combined with dual-energy mammography, is used to increase the diagnostic accuracy of full-field digital mammography (FFDM). The first commercially available FDA approved CEM system was introduced by GE Healthcare in 2011^1^. The use of CEM in the breast imaging community is steadily increasing, as is not only reflected by the increasing number of installations and scientific literature, but also by the fact that multiple vendors now have their own commercially available mammography systems capable to perform CEM.

The different CEM capable mammography units are released under different brand names by various vendors. For example, GE Healthcare marketed the name ‘Contrast-Enhanced Spectral Mammography’ (CESM), while both Hologic and Fujifilm christened their techniques ‘Contrast-Enhanced 2D imaging’ (CE2D). Siemens Healthineers released their product under the name ‘Titanium Contrast-Enhanced Mammography’ (TiCEM)^2^.

Studies have shown that CEM is consistently superior to FFDM in terms of diagnostic accuracy, especially in women with dense breasts^3^. Due to this increased diagnostic accuracy, it is even being considered for screening purposes^4^. In a review by Zhu et al., including 18 studies, the pooled sensitivity and specificity of CEM was 0.89 and 0.84, respectively^5^. More recently, Suter et al. published a meta-analysis of eight prospective studies, finding a pooled sensitivity and specificity of 0.85 and 0.77, respectively^6^.

Current knowledge of CEM, however, is mainly based on studies that have used a system from GE Healthcare. Zanardo et al. observed that only 14 of the 84 studies (1755/14012 cases) included in their review were performed on another system^7^. Even then, some of these studies focused on other topics than diagnostic accuracy (e.g., evaluation of disease extent, radiation dose studies, or CEM as response monitoring tool in women treated with neoadjuvant chemotherapy). To facilitate a broader acceptance of CEM in clinical practice, it is important to study the consistency and reproducibility of results, also on other vendors and their systems. However, it is unethical to have women undergo more than one CEM exam solely for this research purpose.

Therefore, the aim of this retrospective study was to evaluate the diagnostic performance of FFDM versus CEM on a Hologic system using three different readers.

## Methods and materials

### Patient population

The CEM system used was installed in January 2019 and the first clinical CEM was performed in February 2019. All consecutive patients who underwent CEM in the period of February to December 2019 were eligible for this retrospective study. Indications for CEM imaging included: additional imaging after screening recall, screening of women with hereditary breast cancer when breast MRI was contra indicated, patients with symptoms which raise suspicion of breast cancer (*e.g.,* palpable masses, skin abnormalities, etc.), follow-up after breast cancer treatment, inconclusive findings after prior breast imaging, supplemental imaging in dense breasts, and coincidental findings in non-breast specific imaging (*e.g.,* chest CT). Excluded were patients who were treated with neoadjuvant chemotherapy, where CEM was solely used as response monitoring tool.

The Medical Research Involving Human Subject Act (WMO) did not apply to this study protocol and an official approval of this study by the Medical Ethics Review Committee of University hospital Maastricht and Maastricht University (METC azM/UM) was not required (decision no. METC 2019-1427). Due to the retrospective nature of the study, the METC azM/UM and the Trial Advisory Committee of Laurentius Hospital (decision no. 20/10) waived the requirement for obtaining informed consent. All methods were carried out in accordance with relevant guidelines and regulations.

### Imaging protocol

The CEM imaging principle and protocol was described earlier^2,8,9^. In short, an iodine based contrast agent is injected intravenously two minutes prior to image acquisition. After two minutes, a dual-energy mammography examination is performed in at least craniocaudal (CC) and mediolateral oblique (MLO) view of each breast. As final result, the radiologist can read a low-energy (LE) image (which is comparable to a standard full-field digital mammogram or FFDM^10,11^) and a recombined (iodine) image (in which areas of contrast uptake can be appreciated). In this study, a non-ionic, low-osmolar monomer, iobitridol (Xenetix 300® (300 mg I/ml), Guerbet) was administered (dose 1.5 mL/kg body weight, flow rate of 2.5–3 mL/s, followed by saline flush) using a power injector (MEDRAD Stellant, Bayer). All CEM exams were performed on a single dedicated mammography system (Selenia Dimensions, with I-View CE2D upgrade, Hologic Inc.).

### Image analysis

Three readers independently assessed all CEM exams on a dedicated workstation (Coronis Fusion 6MP LED monitor, Barco; SecurView workstation, Hologic). All readers had extensive experience reading breast imaging exams, ranging from 11 to 25 years. First, the LE images were assessed and scored using the Breast Imaging Reporting And Data System (BI-RADS) classification^12^. Second, the complete CEM exams (*i.e.,* LE plus recombined images) were assessed. When viewing the complete CEM exam, the radiologists were allowed to adjust their initial BI-RADS classification if deemed necessary. All readers were blinded for each other’s classifications and the final diagnosis. The breast density classification was retrieved from the imaging reports.

### Reference standard to assess true disease status

Histopathology was the gold standard to ascertain true disease status for patients in whom biopsy was performed. The diagnosis of a cyst was confirmed by an eclipse sign on the CEM or by ultrasound-guided aspiration^13^. All histological analysis were performed according to our current national guidelines. The maximum diameter of the breast cancer diagnosed, its phenotype, grade, receptor status, and axillary lymph node status were retrieved from the pathology reports. A minimum of one year follow-up was used in the final analysis as additional reference standard for cases that were considered benign (including cysts) or negative after CEM evaluation, in order to identify any false negative imaging results. If no cancer was diagnosed after this period, the case was definitely considered benign or negative.

### Statistical analysis

Diagnostic parameters including sensitivity, specificity, positive predictive value (PPV), and negative predictive value (NPV) were compared between LE and CEM, separately for the three readers and for all readers combined. BI-RADS 1–3 lesions were considered to be negative or benign, and BI-RADS 4–5 were considered to be malignant. To account for the correlation between multiple observations within patients, the variance and 95% confidence intervals were obtained with a robust variance estimator^14^. A logistic random effects model was used to test the differences in sensitivity and specificity between LE and CEM for statistical significance. Receiver operating characteristics (ROC) curves and the area under the curve (AUC) with corresponding 95% confidence intervals were calculated for both LE images and the complete CEM exam. The difference between the AUC for LE images and CEM was tested for significance using an algorithm suggested by DeLong et al.^15^. Statistical significance was set at *p* values ≤ 0.05. Statistical analyses were performed using SPSS (IBM Corp. version 26) and STATA (StataCorp LLC., version 14).

## Results

### Patient characteristics

A detailed overview of image indications and diagnoses is presented in Table 1. Of the 162 patients who underwent CEM, 152 patients met the inclusion criteria. The ten excluded patients had received CEM to monitor tumor response during neoadjuvant chemotherapy treatment (n = 4) or had incomplete CEM data (n = 6). Most patients (95/152; 62.5%) were referred to the hospital for additional imaging after a recall from the national breast cancer screening program. Mean age was 57.9 years (± SD: 10, range 34–83 years).

**Table 1:** Image indications, final diagnosis of malignant and benign cases.

Patients (n = 152)		Percentage of patients (%)
Age	Years	
Mean	57.9	
Range	34–83	
Image indications	Number of cases	
Hereditary screening	13	8.6
Recall from National screening program	95	62.5
Symptomatic patients	12	7.9
Follow-up	13	8.6
Inconclusive findings	16	10.5
Pre-operative staging	3	2.0
Breast density	Number of cases	
ACR A	8	5.3
ACR B	87	57.2
ACR C	36	23.7
ACR D	8	5.3
Not reported	13	8.6
Malignant diagnosis	Number of cases	
Invasive carcinoma NST	29	82.9
Invasive lobular carcinoma	4	11.4
DCIS	1	2.9
Mucinous carcinoma	1	2.9
Benign diagnosis	Number of cases	
Negative/normal tissue	76	65.0
Negative/lymphoma	2	1.7
Cyst	31	26.5
Fibroadenoma	4	3.4
Fibrosis	1	0.9
Lymph node	3	2.6

*ACR* American College of Radiology, *NST* No special type, *DCIS* Ductal carcinoma in situ.

**Figure 1:**
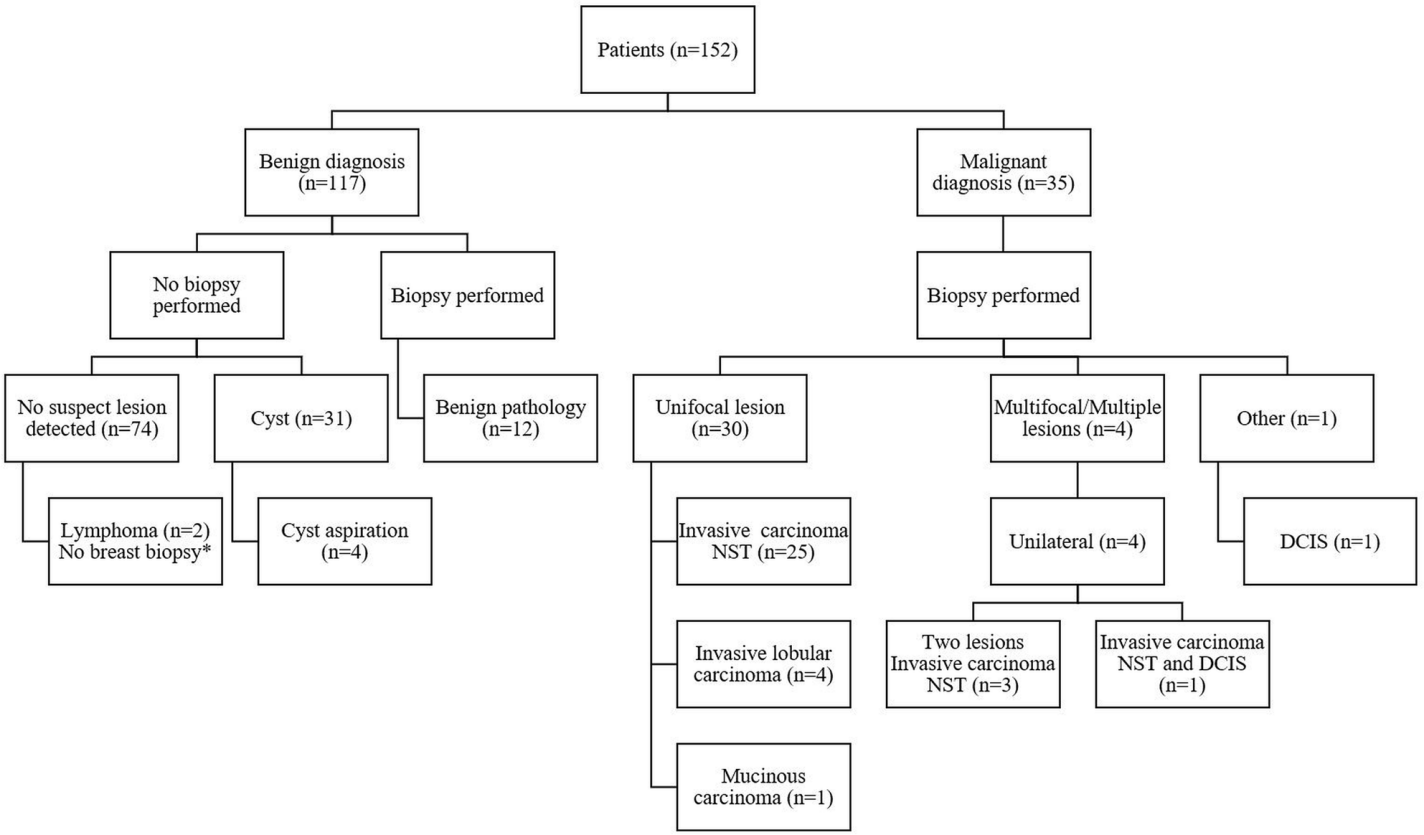
Patient diagnostic flowchart. *Axillary biopsy revealed lymphoma, no suspect lesion on CEM. *NST* No special type, *DCIS* Ductal carcinoma in situ, *CEM* Contrast-enhanced mammography.

The majority of the patients did not have breast cancer: 117 cases (77.0%) were considered benign, of which 74 were negative (48.7%), 31 cases were cysts (20.4%) and in twelve cases biopsy results showed a benign lesion (7.9%) (Table 1). Thirty-five patients were diagnosed with breast cancer (and/or ductal carcinoma in situ (DCIS)), and two patients with lymphoma. These two patients suffered from axillary lymph node enlargement and were considered as negative cases, since no intramammary lesion was detected with CEM. The diagnosis of lymphoma was made based on a palpable mass in the axilla. Figure 1 shows a flow chart of the patients’ diagnostic pathways.

### Tumor characteristics

In the 35 breast cancer and DCIS patients, a total of 39 malignant lesions were detected. The mean tumor diameter of these 39 lesions on CEM was 14.4 mm ± 10.2 (range 4–60 mm). In four patients, two malignant lesions were detected within the same breast. Histopathology confirmed that in three of these four patients the multifocal lesions were identical to the primary index tumor concerning subtype of breast cancer and grade. The fourth patient, had a primary tumor consisting of invasive carcinoma NST with a second lesion being DCIS grade 2. The majority of malignant cases had invasive carcinoma NST (29/35; 82.9%), followed by invasive lobular carcinoma (4/35; 11.4%). Estrogen receptor (ER) status was positive in 31 of the 35 invasive malignancies (88.6%), Progesterone receptor (PR) was positive in 25 malignancies (71.4%), and Human Epidermal growth factor Receptor 2 (HER2/neu receptor) status was positive in seven malignancies (20.0%). In 19 of the 35 malignant cases (54.3%) tumor grade was I, 15 cases (42.9%) were grade II, and one case (2.9%) was grade III. An overview of tumor characteristics is provided in Table 2.

**Table 2:** Tumor characteristics specified per breast cancer subtype, hormonal receptors, and tumor grade.

	Invasive carcinoma NST	Invasive lobular carcinoma	DCIS	Mucinous carcinoma
ER positive	26 (89.7%)	4 (100.0%)	N/A	1 (100.0%)
PR positive	23 (79.3%)	2 (50.0%)	N/A	0 (0.0%)
HER2/neu positive	6 (20.7%)	1 (25.0%)	N/A	0 (0.0%)
Grade I	17 (58.6%)	1 (25.0%)	0 (0.0%)	1 (100.0%)
Grade II	11 (37.9%)	3 (75.0%)	1 (100.0%)	0 (0.0%)
Grade III	1 (3.4%)	0 (0.0%)	0 (0.0%)	0 (0.0%)

*ER* Estrogen receptor, *PR* Progesterone receptor, *HER2/neu* Human epidermal growth factor receptor 2, *NST* No special type, *DCIS* Ductal carcinoma in situ, *N/A* Not available.

All patients underwent treatment for their (pre-) malignancy, the majority with primary surgery (n = 30) and surgery after neo-adjuvant chemotherapy (n = 2). In one case the patient did not undergo surgery and in two other cases the patients were operated elsewhere. The mean tumor diameter of the operated 34 lesions was 12.4 mm ± 6.9 (range 0–29 mm). Axillary lymph node status was pN + in seven cases, pN0 in 24 cases, and not reported or unknown in four cases.

### Diagnostic accuracy

Median follow-up of CEM exams initially classified as benign/negative was 25 months (range 17–28 months). In this period, no additional breast cancer diagnoses were established in these cases, classifying them as definitely benign/negative.

**Figure 2:**
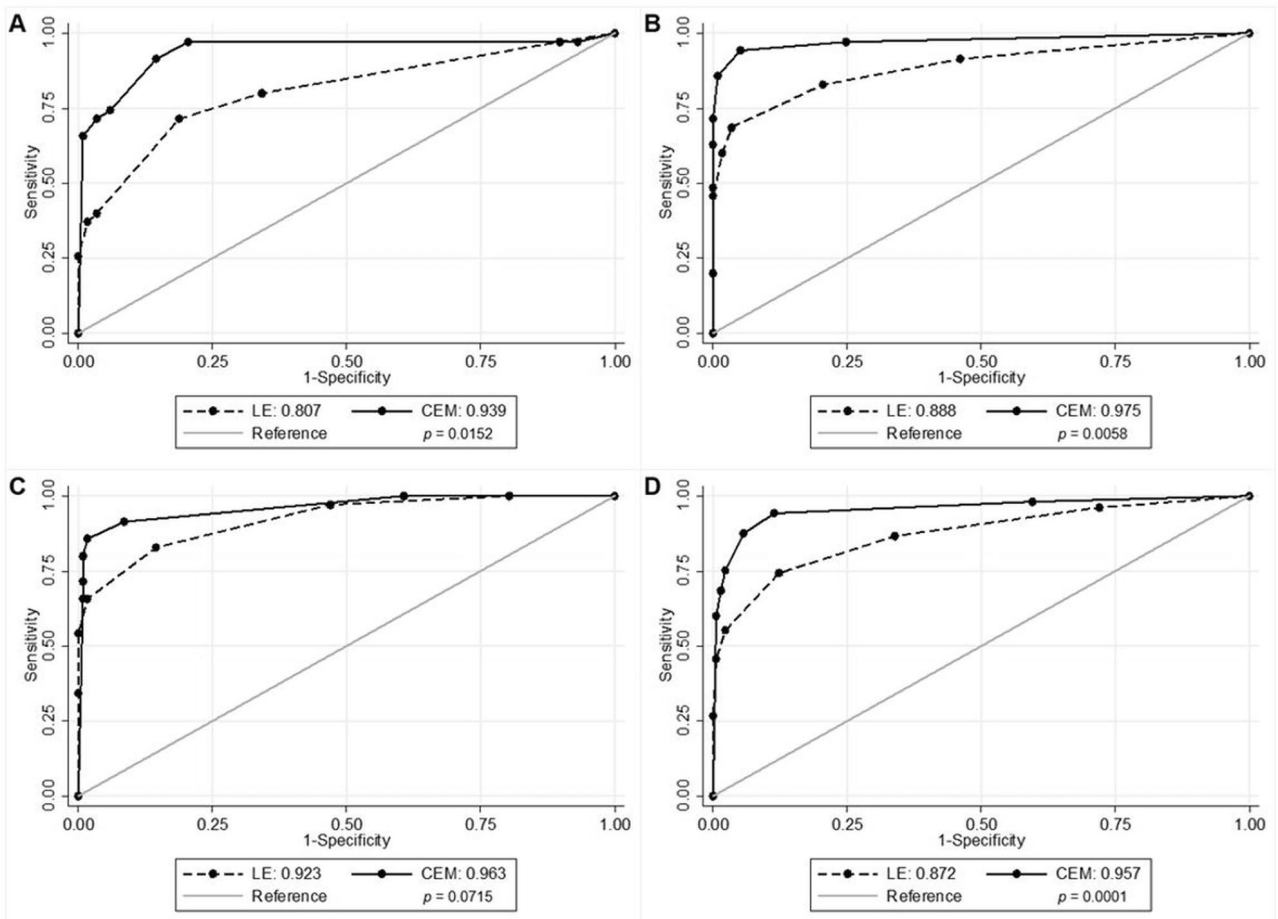
ROC curves and corresponding AUC values for LE and CEM, and *p* values. (**A**) Reader 1; (**B**) Reader 2; (**C**) Reader 3; (**D**) All readers combined. *ROC* Receiver operating characteristics, *AUC* Area under the curve, *CI* Confidence interval, *LE* Low-energy, *CEM* Contrast-enhanced mammography.

CEM exams showed overall higher sensitivity and specificity than LE exams for all three readers. Mean sensitivity for all readers combined increased from 74.3% (LE) to 87.6% (CEM) (*p* < 0.001), while mean specificity increased from 87.8% (LE) to 94.6% (CEM) (*p* = 0.0146). Mean PPV and mean NPV increased from 64.5% to 82.9% and 91.9% to 96.2%, respectively. The AUC increased for all three readers, with statistically significant increases being observed in readers 1 and 2. The AUC of all readers combined increased from 0.872 for LE images to 0.957 for CEM exams (*p* = 0.0001). Table 3 shows the diagnostic accuracy parameters per reader and for all readers combined. Figure 2 and Table 4 provide an overview of the ROC curves and the corresponding AUC values of all readers independently and combined.

**Table 3:** Diagnostic accuracy in percentages of LE and CEM exams per individual reader and for all readers combined.

Reader	Exam	Sensitivity (95% CI) [N/D]	Specificity (95% CI)	PPV (95% CI)	NPV (95% CI)
Reader 1	LE	71.4 (56.2–83.5) [25/35]	81.2 (76.6–84.8) [95/117]	53.2 (41.8–62.2) [25/47]	90.5 (85.4–94.5) [95/105]
	CEM	91.4 (78.2–97.7) [32/35]	85.5 (81.5–87.3) [100/117]	65.3 (55.8–69.8) [32/49]	97.1 (92.6–99.2) [100/103]
Reader 2	LE	68.6 (55.8–76.0) [24/35]	96.6 (92.8–98.8) [113/117]	85.7 (69.7–95.0) [24/28]	91.1 (87.5–93.2) [113/124]
	CEM	85.7 (75.1–88.4) [30/35]	99.1 (96.0–100.0) [116/117]	96.8 (84.8–99.8) [30/31]	95.9 (92.8–96.7) [116/121]
Reader 3	LE	82.9 (68.5–92.3) [29/35]	85.5 (81.2–88.3) [100/117]	63.0 (52.1–70.2) [29/46]	94.3 (89.6–97.5) [100/106]
	CEM	85.7 (74.5–90.4) [30/35]	98.3 (94.9–99.7) [115/117]	93.8 (81.5–98.8) [30/32]	95.8 (92.6–97.2) [115/120]
All readers mean	LE	74.3 (60.3–84.6) [78/105]	87.8 (83.6–91.0) [308/351]	64.5 (52.1–75.2) [78/121]	91.9 (86.1–95.4) [308/335]
	CEM	87.6 (74.3–94.5) [92/105]	94.6 (91.7–96.5) [332/351]	82.9 (72.8–89.8) [92/111]	96.2 (91.5–98.4) [332/345]

The numbers used to calculate the percentages are presented in brackets.

*CI* Confidence interval, *PPV* Positive predictive value, *NPV* Negative predictive values, *LE* Low-energy, *CEM* Contrast-enhanced mammography.

**Table 4:** AUC values and corresponding *p* values for both LE and CEM exams, per individual reader and for all readers combined.

Reader	AUC (95% CI) LE	AUC (95% CI) CEM	*p* values (α = 0.05)
Reader 1	0.807 (0.718–0.897)	0.939 (0.882–0.997)	*p* = 0.0152
Reader 2	0.888 (0.815–0.962)	0.975 (0.939–1.000)	*p* = 0.0058
Reader 3	0.923 (0.872–0.974)	0.963 (0.928–0.997)	*p* = 0.0715
All Readers	0.872 (0.829–0.916)	0.957 (0.932–0.982)	*p* = 0.0001

*AUC* Area under the curve, *CI* Confidence interval, *LE* Low-energy, *CEM* Contrast-enhanced mammography (CEM).

### Discrepancies between readers and diagnosis

**Figure 3:**
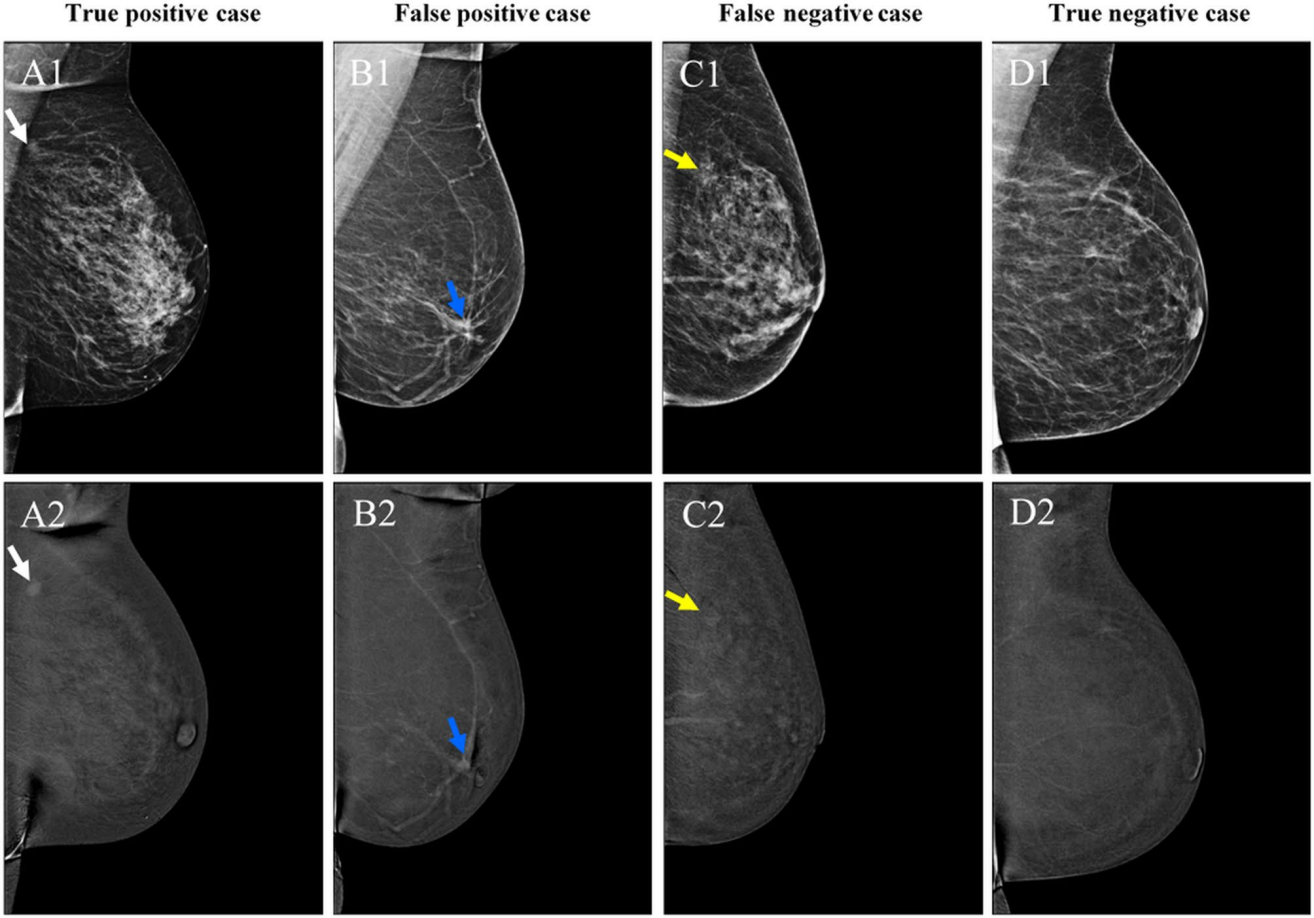
Examples of a true positive (**A**), false positive (**B**), false negative case (**C**), and true negative case (**D**). Top row represents the low-energy images, bottom row represents the recombined images. A1 shows an ill-defined, round mass anterior to the pectoral muscle (white arrow). The mass shows enhancement (A2). In B1, an ill-defined mass can be observed in the retro areolar zone with spiculated margins, also showing enhancement on the recombined images (B2, blue arrow). In C1 and C2, none of the radiologists classified this case as malignant, although a cancer was present as a subtle, ill-defined focal asymmetry at the site of the yellow arrow. The distortion did not show any enhancement. Finally, D1 and D2 show a negative case (no abnormalities or focal enhancement visible in the breast). However, this patient suffered from an axillary lymphoma and was therefore classified as ‘negative’, as no breast cancer was detected. Histopathological results from **A**, **B** and **C** were invasive carcinoma of no special type, fibroglandular tissue, and invasive lobular carcinoma, respectively.

None of the 152 cases was scored false positive on CEM by all three readers. Eighteen cases were scored as false positive on CEM by one reader and one case by two readers. Two cancer diagnoses were overlooked on CEM by all readers: one case of invasive lobular carcinoma, and one case of mucinous carcinoma, with tumor diameters of 1.0 cm and 0.7 cm, respectively. This case of invasive lobular carcinoma was also missed by all readers on LE, while one reader did detect the mucinous carcinoma on LE. In clinical practice the missed mucinous carcinoma was detected on CEM by the radiologist on duty, while the missed invasive lobular carcinoma was detected by additional breast MRI. Figure 3 shows an example of a true positive case, true negative case, false positive case, and a false negative case.

## Discussion

CEM is an emerging breast imaging modality, introduced in 2011 by GE Healthcare, but is currently being offered by four vendors as commercial systems^2^. Many studies have shown that CEM’s diagnostic accuracy is superior to FFDM, even matching the performance of breast MRI^16–19^. However, these results are mainly based on studies performed on GE Healthcare systems. In this study, we demonstrated that an increase in diagnostic performance can also be achieved with a Hologic system when compared to FFDM. When using Hologic CEM, sensitivity significantly increased from 74.3 to 87.6%, and specificity from 87.8 to 94.6%.

Although several reviews and meta-analyses have been published^5,6,20^, we believe that the one published by Zhu et al. is the most comprehensive one. In our opinion, their review provided the most extensive and up-to-date overview, including 18 studies published between 2014 and 2017. Seventeen of the 18 studies were performed on systems by GE Healthcare and only one study on a Hologic system. No studies using equipment of other vendors were included. In this review, the pooled sensitivity and specificity for CEM was 89% and 84%, respectively. The AUC value based on their summary ROC curve was 0.957. The combination of sensitivity and specificity observed in our study falls within the 95% confidence interval of the summary ROC curve given by Zhu et al.^5^. Based on these findings, we concluded that sensitivity and specificity for CEM on a Hologic system are in line with prior findings for sensitivity and specificity of CEM studies that were mainly performed on GE Healthcare equipment.

Reproducibility is one of the pillars in science. In clinical studies, this means that certain results obtained can be achieved again (‘reproduced’) when the study is repeated with a similar methodology but by other, independent researchers. Reproducibility is important, since no claim can be proven by a single published study. Despite the utmost efforts of investigators to perform a study to the best of their ability, reproducible results provide the scientific community the only true transparency in results, giving us confidence in the results as well. In the context of the current study, it is important to reproduce the claims of CEM superiority over FFDM mainly made on GE Healthcare equipment, as this creates confidence that the results stand even when other equipment is used.

The number of studies using Hologic CEM units is still limited, often focusing on equally important aspects of CEM, such as studies on radiation exposure or evaluation of disease extent. To the best of our knowledge, there are currently eleven studies published that assessed diagnostic accuracy^18,19,21–29^. However, these studies often stated only partial diagnostic performance, for example only sensitivity. These studies were also relatively small, with sample sizes varying from 34 to 100 patients, with two exceptions up to 208 and 326 patients. One study provided a case-by-case description of the performance of CEM^26^, but in the other ten studies, sensitivity of CEM ranged from 81.5 to 100%^18,19,21–25,27–29^, with specificity available in just three studies and ranging from 42.6 to 79.6%^27–29^. However, the differences in methodologies used in these studies hamper a robust comparison of our results with these eleven studies.

Our study has some limitations. Firstly, the dominating indication for the CEM examinations in this population was the evaluation of recalls from breast cancer screening. However, a sub analysis of these (recalled) women versus all other indications showed that there was no statistically significant difference in sensitivity and specificity (data not shown). Secondly, the follow-up period used to determine the true negative disease status of cases deemed to be benign or negative could be considered to be short. A follow-up period of at least two years is preferable, which is a commonly accepted time interval used in screening programs. However, the follow-up period used was considered the *minimum* time interval. Half of the cases had a follow-up period of more than two years, while the shortest follow-up period lasted 17 months. In addition, the prevalence of interval carcinomas is low (0.21%), rendering it unlikely that an extra year of follow-up would reveal a substantial number of missed cancer cases^30^. Consequently, prolonging follow-up will not result in significant alterations of the study conclusion.

In summary, this study showed that CEM performed on a Hologic system improved both sensitivity and specificity when compared to FFDM. Our observed diagnostic performance falls within the 95% CI of summary ROC curves of a previously published meta-analysis. Thus, it can be safely assumed that the type of CEM unit does not seem to be a decisive factor in studies that are using equipment from different vendors.

## Data Availability

The dataset generated and analysed during the current study are available from the corresponding author on reasonable request.
